# The Limitations of Periapical X-ray Assessment in Endodontic Diagnosis—A Systematic Review

**DOI:** 10.3390/jcm12144647

**Published:** 2023-07-12

**Authors:** Alexandru Gliga, Marina Imre, Simone Grandini, Crystal Marruganti, Carlo Gaeta, Dana Bodnar, Bogdan Alexandru Dimitriu, Federico Foschi

**Affiliations:** 1 Department of Operative Dentistry, Faculty of Dental Medicine, “Carol Davila” University of Medicine and Pharmacy, 050474 Bucharest, Romania; dr.alexandrugliga@gmail.com; 2 Unit of Endodontics, Department of Medical Biotechnologies, Periodontology, Restorative and Paediatric Dentistry, University of Siena, 53100 Siena, Italy; simogr@gmail.com (S.G.); marruganti@gmail.com (C.M.); odontoiatriagaeta@libero.it (C.G.); 3 Department of Complete Denture, Faculty of Dental Medicine, “Carol Davila” University of Medicine and Pharmacy, 020021 Bucharest, Romania; melim.marina@gmail.com; 4 Department of Endodontology, Faculty of Dental Medicine, “Carol Davila” University of Medicine and Pharmacy, 050474 Bucharest, Romania; 5 Faculty of Dentistry, Oral and Craniofacial Sciences, King’s College London, London SE19RT, UK; 6 Peninsula Dental School, University of Plymouth, Plymouth PL6 8BT, UK

**Keywords:** apical periodontitis, cyst, endodontic diagnosis, granuloma, histology periapical radiology

## Abstract

Diagnosis is a key aspect in endodontic treatment, in a decade where invasive interventions are misapprehended as social tendency instead of medical necessity. All diagnostic facets should be considered before intending the operative phase. Intraoral endodontic radiology-based diagnosis has been shown to be limited. Periapical X-ray is the most used endodontic imaging, yet it does not provide high accuracy. Traditionally, dentists have been trained to diagnose a cyst by certain aspects (size, shape and appearance); hence, an assumption that teeth are affected by “periapical cyst” were subjected to unnecessary extraction or apicoectomy. The aim of this systematic review is to critically appraise the publications that relate the histological diagnosis of a periapical lesion (considered the gold standard) to intraoral X-ray investigation. Ovid Medline, PubMed, ScienceDirect, Mendeley and Scopus were searched for English-language studies comparing periapical diagnosis obtained by using two techniques (histopathology and X-ray). Sixteen articles were included for the final analysis (qualitative and quantitative evaluation) out of which only two supported the statement that periapical diagnosis can be coherently assessed through periapical imaging. Although there is not enough evidence to deliver a definitive conclusion, there are many publications that refute the diagnosis of a cyst via periapical X-ray.

## 1. Introduction

Diagnosis in Endodontology can be a somewhat controversial subject, especially when it comes to decision making and a proper clinical attitude [1,2]. Reversible or irreversible pulpitis, cyst or periapical inflammation are the daily practice self-reflections of many clinicians [2,3]. Pulp diagnosis and periapical diagnosis are subject to interpretation, knowledge, experience, state of mind and other aspects that convert the assessment protocol into a personal perception [4]. The reason for performing any medical procedure should be based on a defined assessment checklist that has to be thoroughly completed before embarking on any irreversible therapy [5]. Standardisation and clear protocols development as well as coherent terminology and classifications help clinicians to minimise not only the subjectivity in reaching a diagnosis, but also the risk of overtreatment. As a part of scientific development, established dogmas were proved as fallacy and are now redundant. It stands to reason that technology will develop and current conventional wisdom and ‘facts’ will become obsolete [6].

Teeth with reversible pulp inflammation may undergo unnecessary root canal treatments; similarly, periapical lesions may be considered to require extractions due to an improper diagnosis of refractory cyst [4,7,8].

Even though many General Dental Practitioners and Endodontists are well trained, in some cases their decisions are occasionally governed by one specific imaging characteristic and/or by the result of a single diagnostic tool that has been proven to have low specificity and sensitivity [7,9].

The physiological or pathological status of the periapical tissue is assessed by histology as a gold standard. In practice, imaging tools are used to infer the histological status. A histological result can be subjected to debate due to various collection methodologies available as well as different techniques for the preparation steps of the analysed sample or even because of the histological terminology. Serial sectioning and sample alignment are considered to be an elective procedure, more accurate than step sectioning, having an important impact on the final histopathological diagnosis [10]. The assessment of serial layers of a biological sample can offer the examiner a three-dimensional perception; hence, the histopathological diagnosis is more specific, although more time consuming.

When it comes to imaging, contrast, the shades of grey, filters, artefacts, parallelism, voxel/pixel size, etc., are just a few variables that can lead to a misguided approach [11,12,13,14].

It is of great importance to know the capacity of periapical X-ray and to pragmatically assess the nature of a periapical lesion. However, trying to determine a differential diagnosis of cyst, granuloma or epithelium cells on a periapical X-ray might be an assumption.

A consensus has been reached that a periapical cyst follows chronic apical periodontitis [15]. One of the most common arguments for the ability to diagnose a cyst by a periapical X-ray is the observation of the epithelial lining that defines clear margins for the radiolucent area. Even if to some extent this statement could be true, it is known that even if 52% of periapical lesions contain organised epithelium cells, still the incidence of cystic lesions is 15% [15]. This number confirms the 85% success rate in orthograde retreatments as the periapical cyst might only be managed through retrograde endodontic approach [16]. Taking this into consideration and the fact that assessing the presence of epithelial cells on a periapical X-ray can appear over-elaborated due to imaging dissimilarities, it is clear that the ability of such differential diagnosis is questionable. Moreover, considering that a less invasive approach should be opted in any case (when possible), clinicians should not perform surgery unless it is the least invasive option available. All these aspects clearly indicate that, for the moment, the assessment and treatment plan for cases with persistent periapical pathology is “retreat and follow-up”. In most situations, non-surgical endodontic treatment is achievable and may represent not only the least invasive option, but also a feasible approach with a highly predictable outcome [17,18,19,20,21].

This review does not disprove the importance or the necessity of pre-, intra- and post- periapical X-ray evaluation, but highlights the limitations of this clinical investigation. It is mandatory for clinicians to know to what extent a tool can be trusted in order to use it properly. Periapical X-ray is a two-dimensional image formed after projecting a three-dimensional anatomy where details can be superimposed [22]. Even though CBCT scan offers a new perspective on oral imaging exploration, it should be performed only in cases where further investigation is needed, nevertheless considering its own limitations.

The presence or absence of lamina dura can be assessed through periapical X-ray, but there is no correlation found between this aspect and the histological diagnosis of a cyst [15]. Furthermore, the dimension of the lesion was subject of a study, but its correlation with the gold standard was weak even for CBCT investigation; thus, biopsy analysis was advised [23].

Density measurements are not able to deliver a feasible and reliable result that can be linked predictably to histological aspects [24]. Another study reviewing 104 cases of periapical lesions could not match any common aspects between periapical X-ray and histology in order to develop a diagnostic pattern [25]. Another retrospective study calls for advance investigation each time there is uncertainty in approaching a case to avoid misdiagnosis and unnecessary treatments [26].

The aim of this review is to compare the histological diagnosis of apical cyst to periapical X-ray characteristics based on data found in high-quality studies.

## 2. Materials and Methods

This systematic review was registered in PROSPERO (number CRD42023406854) and followed the PRISMA (Preferred Reporting Items for Systematic reviews and Meta-Analyses) guidelines [9] using the PICO (Population, Intervention, Comparison, Outcome) framework to investigate the following clinical questions: “To what extent is a clinician able to diagnose a periapical pathology using periapical imaging investigation and how would this detailed assessment impact the clinical approach?”. P = Patients with apical periodontitis. I = Periapical X-ray diagnosis. C = Histological examination. O = Correlation between the X-ray and the histological findings to avoid misdiagnosis and overtreatment.

Five electronic databases were searched: Ovid Medline, PubMed, ScienceDirect, Mendeley and Scopus. The search methodology applied Boolean operators (AND, OR) for the following key words: “periapical periodontitis” AND (X-ray OR Radiography) AND “diagnosis” AND “histology”. The search results were narrowed down by subject, abstract and methodology.

All articles selected were read to decide if they met the inclusion criteria. The quality of these studies was assessed before the articles’ final selection (clear goal, detailed methodology, etc.).

### 2.1. Inclusion Criteria

The following aspects were searched for inclusion:

1. Articles that are published in English or use English as a second language;
2. Articles that use histopathology as a gold standard for periapical diagnosis;
3. Articles that have used periapical X-ray as the main imaging technique or that include periapical X-ray as a comparative study to other imaging investigations;
4. Articles that perform study on living organisms: human beings or animals;
5. Articles that relate the histopathological diagnosis to the imaging investigation;
6. Studies that approach distinctly the imaging assessment (if the assessment has been performed using various techniques—OPG, periapical, CBCT).

### 2.2. Exclusion Criteria

The exclusion principles were the following:

1. Studies performed on human or animal cadavers (simulated tissue for X-ray exposure is not an accurate assessment);
2. Studies that do not use histopathology as a gold standard in diagnosis;
3. Book chapters, personal opinions, letters, narratives, commentaries and conference abstracts;
4. Studies that could not be found published in English;
5. Studies that use other imaging techniques to assess the periapical status (OPG/CBCT);
6. Induced pathology was not accepted (intra-oral exposed pulp for a defined period of time).

### 2.3. Study Selection

Titles and abstracts were independently screened for relevance by two calibrated reviewers (un-weighted Cohen’s *k* score of 0.90) (A.G., C.G.). Subsequently, the pre-selected articles were screened for full-text analysis by both reviewers according to the eligibility criteria. Any disagreement at any stage (title/abstract or full text) was resolved through discussion with a third author (C.M.) in order to reach consensus.

### 2.4. Data Collection Process

**Figure 1:**
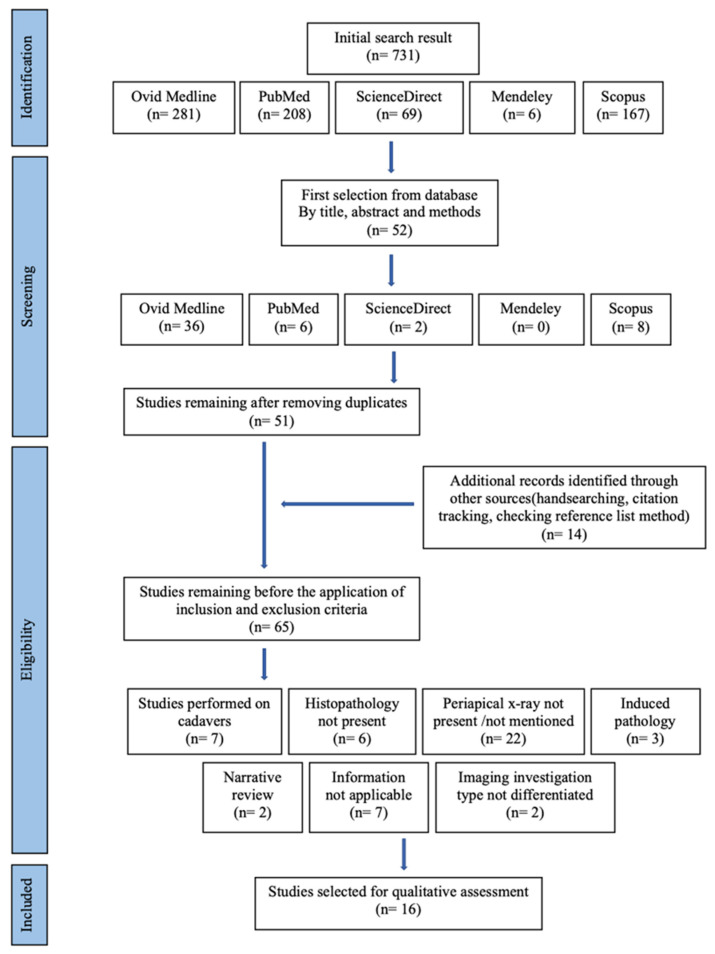
Flowchart of the search strategy indicating the number of articles and source.

Data was collected by a datasheet by two independent reviewers (A.G., C.G.) during full-text analysis. ProQuest RefWorks software was used to find and remove duplicates. If one of the exclusion criteria was found during the full-text reading, the study was considered ineligible. If no exclusion criteria was found, the article was fully read to assess the bias risk and the quality of the research. Articles that did not appear to meet the inclusion criteria or had poor methodology specification or unclear objective were excluded. A schematic of this process is given in Figure 1.

## 3. Results

Fifty-two articles were included in final evaluation, one was removed because it was a duplicate. After the full-text reading of the 51 articles, 14 more articles derived from extended searching strategies were added and a total of 65 studies were included for review. Forty-nine publications were eliminated due to the following reasons: 22 were excluded because the imaging technique used for assessment was not periapical X-ray, while only nine studies, out of which two were narrative reviews, did not provide applicable information for the ongoing review. Seven studies were performed on cadavers, induced periapical pathology was found in three studies and two were excluded for not differentiating between the types of imaging used in the research. Studies that were captured during the initial search, but excluded upon application of inclusion and exclusion criteria are given in Table 1.

Finally, 16 studies that matched the inclusion and exclusion criteria were found. A brief summary of included studies in this review is listed in Table 2.

For each selected article, a quality score was calculated using an adapted table (Table 3) from McGrath et al. (2009) [27]. Any disagreement between investigators has been solved through consensus panel. The results for the quality assessment ranged between 11 and 20.5 and the highest score was recorded as previously demonstrated [28].

**Table 1:** Table containing the 49 excluded articles after full-text reading.

Reason for Exclusion	Article (Author and Year)
The study was performed on cadavers and/or used simulated tissue for X-ray exposure	(Trope et al., 1989) (Green et al., 1997) (Barthel, Zimmer and Trope, 2004) (Kanagasingam, Hussaini et al., 2017) (Leonardi Dutra et al., 2016) (Kanagasingam, Lim et al., 2017) (Holtzmann et al., 1998) [29,30,31,32,33,34,35]
The study did not use histopathology as a gold standard method for diagnosis confirmation	(Estrela et al., 2008) (Tsai et al., 2012) (Low et al., 2008) (Halse, Molven and Fristad, 2002) (Lofthag-Hansen et al., 2007) (Tikku et al., 2010) [36,37,38,39,40,41]
The study did not use periapical X-ray for imaging assessment or did not clearly mention the X-ray type included in the Materials and Methods section or elsewhere in the article text (OPG/CBCT)	(Lin, Louis M. et al., 1991) (Becconsall-Ryan, Tong and Love, 2010) (Carrillo, Celia et al., 2008) (Simon et al., 2006) (Cotti et al., 2003) (Natkin, Oswald and Carnes, 1984) (Rud, Andreasen, 1972) (Seltzer, S., Bender, Smith, Freedman and Nazimov, 1967a) (Seltzer, S., Bender, Smith, Freedman and Nazimov, 1967b) (Yanagisawa, 1980) (Akinyamoju, O Gbadebo and Adeyemi, 2014) (Penarrocha et al., 2011) (Schulz et al., 2009) (Carrillo, C. et al., 2008) (Block et al., 1976) (Baumann, Rossman, 1956) (Bhaskar, 1966) (Lalonde, 1970) (Kizil, Energin, 1990) (Linenberg, Waldron and DeLaune, 1964) (Alotaibi et al., 2020) [25,42,43,44,45,46,47,48,49,50,51,52,53,54,55,56,57,58,59,60,61]
The study induced the pathological status of the periapical tissue	(Tanomaru-Filho et al., 2009) (López et al., 2014) (Paula-Silva et al., 2009) [22,62,63]
The study is a narrative review	(Hamood, 2001) (Lin, L. M., Huang and Rosenberg, 2007) [64,65]
The study does not contain information that could be linked to the reviewed subject	(Spatafore et al., 1990) (Teixeira-Salum et al., 2010) (Seltzer, Samuel, 1999) (Gallego Romero et al., 2002) (Ricucci, D., Lin and Spangberg, 2009) (Laux et al., 2000) (Patel et al., 2009) [66,67,68,69,70,71,72]
The study did not differentiate between OPG, CBCT or periapical X-ray when performing the imaging analysis and its correlation to the histological status	(Croitoru et al., 2016) (Syrjänen et al., 1982) [73,74]

**Table 2:** Brief description of the included studies. The following table (Table 3) shows the quality assessment procedure applied to the included studies using a number of 19 template questions about standard quality aspects.

Study Selected for Review	Type of Study Perfomed	Sample Size	Details Abouth the PA X-ray Assessment	Details Abouth the Histological Assessment	Results
(O Gbadebo, Akinyamoju and Sulaiman, 2014) [26]	Retrospective study	19 patients	Diagnosis verified by Endodontic Consultants	Results verified by Oral Pathologists	Quantitative measures and quantitative analysis
(Kruse et al., 2017) [9]	Follow-up study	19 patients/19 teeth	Imaging assessment was performed by three observers (two Endodontists, one Oral Radiologist), and when the result had three different versions, a single result was agreed by consesus	Samples were cut at 3–4 µm and analysed by one Oral Pathologist	Quantitative measures and quantitative analysis
(Bornstein et al., 2015) [23]	Follow-up study	62 patients/62 teeth	Imaging assessment was performed by four blinded observers (two Oral Surgeons and two Residents in Oral Surgery)	Two experienced investigators diagnosed the samples, and the disagreement was solved by debate and consensus	Quantitative measures and quantitative analysis
(Berar et al., 2016) [75]	Case study	60 patients/60 teeth	PA X-rays were assessed by two observers	Perfomed using a Leica DM750 Microscope	Quantitative measures and quantitative analysis
(Rózyło-Kalinowska, 2007) [24]	Retrospective study	221 digital X-rays	Digora ver. 2.0 (Soredex—Orion Company, Finland), Dimaxis ver. 2.4.4 (Planmeca, Finland) and Emago ver. 3.42 (Oral Diagnostic Systems, ACTA, Holland) were the software used for density measurements	Not mentioned	Quantitative measures and quantitative analysis
(Shrout, Hall and Hildebolt, 1993) [76]	Case study	10 biopsies of periapical lesions	Regions of interest were digitaly drawn on each X-ray for histogram analysis and cumulative percent histogram calculation	Samples were assessed by two board-certified Oral Pathologists	Quantitative measures and quantitative analysis
(White et al., 1994) [77]	Case study	55 periapical lesions were histologicaly examined	NIH Image was used for X-ray measurements	Histology was assessed by one to three board-certified Oral Pathologist(s)	Quantitative measures and quantitative analysis
(Correa et al., 2017) [78]	Descriptive study	14 samples of apical lesions	VixWin Platinum (version 3.3, Gendex INC, USA) was used for the imaging measurements	Not mentioned	Quantitative measures and quantitative analysis
(Çalışkan et al., 2016) [28]	Retrospective study	93 teeth	Two blinded, trained observers investigated the X-rays in special conditions	Serial sectioning was performed in 4 µm thickness and examined by 1 Oral Pathologist	Quantitative measures and quantitative analysis
(Ricucci, Mannocci and Pitt Ford, 2006) [15]	Case study	57 teeth	Two blinded, trained observers investigated the X-rays in special conditions	Serial sectioning was performed (150–600) in 4–5 µm thickness	Quantitative measures and quantitative analysis
(Ricucci, Siqueira, 2010) [79]	Retrospective study	71 samples	Lesions were divided into 2: ≤5 mm and >5 mm, and no other information was available	Serial sectioning was performed in 4–5 µm thickness, and the assessment was separately performed by two evaluators	Quantitative measures and quantitative analysis
(Priebe, Lazansky and Wuehrmann, 1954) [80]	Prospective study	101 patients	The ø of the apical rarefaction was ≲1 cm, and the imaging assessment was performed independently by four observers (two Oral Surgery Teachers and two Dental Roentgenology Teachers)	Microscopic serial sectioning was performed obtaining > 16,000 sections assessed by a Pathologist	Quantitative measures and quantitative analysis
(Gundappa, Ng and Whaites, 2006) [81]	Comparative in vivo pilot study	15 patients	Three observers (two expert dental Radiologists and one Endodontist) examined the images on day 1, day 7 and day 14 to minimise errors	The biopsies were processed for routine histopathological assessment	Quantitative measures and quantitative analysis
(Mortensen, Winther and Birn, 1970) [82]	Research article	396 periapical lesions were histologically examined	All X-rays were reassessed by one of the authors to minimise subjectivity	The specimens were processed for routine histologic assessment	Quantitative measures and quantitative analysis
(Zain, Roswati and Ismail, 1989a) [83]	Retrospective study	69 cases	The measurements of the radiolucency were performed by operators respecting a defined criteria and a standard protocol	The lesions were reassessed respecting the general criteria	Quantitative measures and quantitative analysis
(Cunningham, Penick, 1968) [84]	Cross-sectional study	41 lesions	The roentgenograms were assessed by two investigators after the injection of the contrast agent	The specimens were examined by an Oral Pathologist	Qualtitative measures and qualtitative analysis

**Table 3:** Quantitative assessment of the quality of the studies that were included in this review.

STUDIES	(Kruse et al., 2017) [9]	(Ricucci, Mannocci & Pitt Ford, 2006) [15]	(Bornstein et al., 2015) [23]	(Rózyło-Kalinowska, 2007) [24]	(O Gbadebo, Akinyamoju & Sulaiman, 2014) [26]	(Çalışkan et al., 2016) [28]	(Berar et al., 2016) [75]	(Shrout, Hall & Hildebolt, 1993) [76]	(White et al., 1994) [77]	(Correa et al., 2017) [78]	(Ricucci, Siqueira, 2010) [79]	(Priebe, Lazansky & Wuehrmann, 1954) [80]	(Gundappa, Ng & Whaites, 2006) [81]	(Mortensen, Winther & Birn, 1970) [82]	(Zain, Roswati & Ismail, 1989) [83]	(Cunningham, Penick, 1968) [84]
**Quality measures**																
Was the research objective clear?	Y	Y	Y	Y	Y	Y	Y	Y	Y	Y	Y	Y	Y	Y	Y	Y
Was the methodology described in detail?	Y	Y	Y	Y	Y	Y	Y	Y	Y	Y	Y	N	Y	N	N	Y
Was the histology assessment protocol described?	Y	Y	Y	N	N	Y	Y	Y	N	N	Y	Y	Y	Y	Y	N
Was the imaging assessment protocol described?	Y	Y	Y	Y	N	Y	Y	Y	Y	Y	N	N	Y	Y	Y	Y
Was it stated how subjects were attained?	Y	Y	Y	Y	Y	Y	Y	Y	Y	Y	Y	Y	Y	Y	Y	Y
Were the subjects clearly defined?	Y	Y	Y	N	Y	Y	Y	N	Y	Y	Y	Y	Y	Y	Y	Y
Was the method of allocation, or similarity between groups described?	N/A	Y	N/A	N/A	N/A	N/A	N/A	N/A	N/A	N/A	Y	N/A	N/A	N/A	N/A	N/A
Were diagnostic tools compared on any variables?	Y	Y	Y	N	Y	Y	Y	Y	N	Y	Y	Y	Y	Y	Y	N
Were the outcome measures clearly defined?	Y	Y	Y	Y	Y	Y	Y	Y	Y	Y	Y	Y	Y	Y	Y	N
Were the outcome measures objective?	Y	Y	Y	Y	Y	Y	Y	Y	Y	Y	Y	Y	Y	Y	Y	N/A
Were the outcome assessors blinded?	Y	N	Y	N	N	N/A	N/A	N	N	N	N	N	N	N	N	Y
Were the participants blinded?	N/A	N	N/A	N/A	N/A	N/A	N/A	N/A	N	N	N	N/A	N/A	N	N/A	N
Was the statistical analysis appropriate?	Y	Y	Y	Y	Y	Y	Y	N/A	Y	Y	Y	Y	Y	Y	Y	N/A
Was the sample size for each group given?	Y	Y	Y	Y	Y	Y	Y	N	Y	Y	Y	Y	Y	Y	Y	N/A
Was there a sample size justification?	N/A	N	N/A	N	N	N/A	N/A	N/A	N	N	N	N	N	Y	Y	N/A
Was the statistical significance defined?	Y	Y	Y	Y	Y	Y	Y	N	N	Y	Y	N	Y	Y	N	N/A
Was drop-out rate given?	Y	N/A	N/A	N/A	N/A	N/A	N/A	N/A	N/A	N	N/A	N	N/A	Y	N/A	N/A
Was drop-out rate <10%?	Y	N/A	N/A	N/A	N/A	N/A	N/A	N/A	N/A	N/A	N/A	N/A	N/A	N	N/A	N/A
Were drop-outs accounted for?	Y	N/A	N/A	N/A	N/A	N/A	N/A	N/A	N/A	N/A	N/A	N/A	N/A	Y	N/A	N/A
**Quality score**	**17**	**14.5**	**15**	**11.5**	**12.5**	**20.5**	**13.5**	**11.5**	**11**	**12.5**	**13.5**	**11**	**15.5**	**14.5**	**13.5**	**10.5**

Summary of points awarded to each study for standard quality aspects of a paper. Each item received equal weighting (Y = 1 point, N = 0 points, N/A = not applicable = 0.5 points), and the overall score was used as a quality index for that study.

The difficulty in conducting a perfectly designed clinical study is well known; thus, a critical overview of the included studies has been applied for delivering a more objective and unbiased study with valuable conclusions.

A 22-year retrospective study was conducted at the University College Hospital Ibadan [26]. The aim of the study was to measure the sensitivity and specificity of conventional radiography on detecting periapical cysts. The number of cases included in the study was limited (*n* = 19); thus, results are not statistically significant. Even though the authors claim a *p* value = 0.003, calculating the Margin of Error (MOE) for a sample size this small and considering a Confidence Level of 95% will result in a MOE of ±22% that does not suggest a good level of precision. Another limitation of this study is that no details on the process of X-ray evaluation are described, such as the number of Endodontic Consultants that were engaged or the technical conditions of the examination. The histological data were analysed by Oral Pathologists, but there were no details on the periapical surgical procedures nor the histological process. Even though the sample size is very small, the conclusions were similar to others cited in the literature [22,85]. Most cases (68.4%) were diagnosed as cyst upon X-ray, but the histology proved that only 15.6% were cysts, while the rest were granulomas [26].

Specificity and sensitivity were also measured in a research article published in 2017 [9]. This study tests the validity of the periapical X-rays and CBCT assessments in cases where apicoectomy was performed and histological diagnosis was conducted due to persistent pathology. Clinical, radiographic, surgical and histological procedures were described in detail. However, the limitations of the study are as follows: no information about serial sectioning in histological assessments and the final sample size included in the study is *n* = 19. The histological diagnosis considered was the level of inflammation of the periapical tissue (categories were no signs of inflammation, mild inflammation or intense inflammation) and in 63% of cases, the category was correctly related to the periapical X-ray assessment [9]. The conclusions drawn suggest that further research is needed and that periapical X-rays as well as CBCT information should be mindfully considered when delivering a diagnosis.

Published in 2015, the next article [23] aimed to connect the histological diagnoses with periapical X-rays and CBCT investigations. The materials and methods were described in detail, even though there are some limitations to be described. The final *n* included was 62, and no serial section technique was mentioned for the histopathological diagnosis. The results demonstrated weak correlation between periapical X-ray and histologic diagnosis (kappa = 0.104), and, although a more precise investigation, the CBCT overestimated the diagnosis of radicular cyst to a value of 8% higher than 2D. The authors assume that the assessment of periapical cyst using periapical X-ray can only be considered “tentative”.

The next study [75] investigated the relationship between the imaging findings and the histological level of inflammation of the periapical tissue of 60 patients with 60 teeth presenting periapical pathology. The paper clearly presents the process of the periapical X-ray examination, the process of tissue sampling and the histological processing. Its main limitations are represented by the fact that the tissue examined was obtained only by alveolar curettage (only soft tissue) and the histological processing was not performed through serial sections. The radiological findings describe 45% of the periapical lesions as granulomas and 55% as cysts, meanwhile the histological assessment of the pathology reports 81.6% granulomas and 18.3% cysts. No conclusions were drawn regarding the percentages described above.

A study published in 2007 [24] analysed the application of periapical radiography on developing differential diagnosis between granulomas and cysts, analysing 221 digital X-rays. Optical density measurements were carried out on the periapical X-rays using digital software and indexes like the average mean density and the difference between the maximum and the minimal density were calculated. The limitations of this research consist in lack of information about the histological analysis (the preservation of specimens, preparation, manipulation and embedding protocol and the assessment of the histopathological diagnosis) and the surgery protocol in collecting the studied samples. The results indicated that agreement between the imaging radiogram and the histopathological diagnosis was around 60%. The author concludes that the possibilities of distinguishing between cysts and granulomas with the use of density indexes is plausible to some extent. However, this imagistic evaluation is not always possible due to anatomical superimpositions; therefore, this technique is not applicable to all cases.

The aim of the study by Shrout and Hildebolt was to agree or disagree upon the capacity of digital radiometric analysis to discern between two periapical pathologies (cysts and granulomas) [76]. The study was performed on tissue specimens sent by Practitioners to the Medical College of Georgia’s School of Dentistry. The Oral Pathology Department analysed the samples, and for the ones diagnosed as granulomas or cysts, X-ray films were requested. The radiographic details of processing, analysis and diagnosis were described; however, important limitations of the study were missing. There was no information on the sample collection process (how the apicoectomy was performed) and no technical data on the histopathological protocol followed, and the *n* was represented by only 10 lesions included exclusively from the posterior part of the lower jaw bone as it is stated in the results section. The conclusions suggest it could be realistic to use radiometric analysis to assess the periapical diagnosis; however, the visual accuracy (no superimpositions) is a decisive factor.

A study by White contradicts the above study by Shrout et al., concluding that there is no differentiation in radiometric measurements between granulomas and cysts [77]. Even though the sample size of this study is larger than the latter study [76] and the methods used for contrast and density settings of the examined images were standardised, recurrent shortcomings must be presented. Histopathology sample collection, processing and analysis are not discussed. This is important because in order for the validity of a histopathological process to be assessed, a defined protocol should be followed. Fifty-five periapical lesions were histologically examined from which 15 were cysts and 40 were periapical granulomas [77]. The results indicated minor distinctions in the acquired values (*p* = 0.530), and the only considerable outcome was that cysts diagnosed lesions tend to be larger in size than granulomas. The authors’ conclusion was that the median grey level does not change considerably between the two diagnoses and that Shrout et al. findings are combatable; hence, imaging assessment remains duplicitous.

A comparative descriptive study was published in 2017 [78], evaluating the correlation of histopathology diagnosis and clinical and radiographical findings. Methods and materials were described in detail, but key aspects regarding the histopathological analysis were omitted. The researchers included a macroscopic diagnosis, a description of which was included in the histopathological chapter where details such as *n*, sample size and principles of histopathological assessment were not present. The *n* does appear later in the article, but after the results and discussion chapter depicted in the first table (Table 1). Another limitation of the article is the number of samples examined, *n* = 14, and the surgical procedure of harvesting them (soft tissue only). The results were similar to other published materials, the prevalence of periapical cyst was 28.5%, no data on the correlation between X-ray diagnosis and histopathological assessment was recorded. An important aspect to be considered is that even though all the X-rays were calibrated and the intention was to use settings as accurate as possible (digitalization and standardization were performed with WXR700 X-ray Film Reader—KAB Dental Equipment; all X-rays were gauged before assessment), no relationship between the macroscopic and the radiologic size of the lesion was found.

Caliskan et al. published a retrospective study for the period June 2007—December 2014, investigating the connections of radiographic features of persisting periapical radiolucent lesions and their histological diagnosis [28]. The study is coherent and well designed, providing detailed information about the materials and methods used. Ninety-three teeth with persistent periapical pathology after root canal treatment were approached by surgical endodontic treatment completed under surgical microscope. All the cases presented preoperative periapical radiographs performed under standardised settings. One of the few limitations found in this article is that the periapical sample was removed using a conservative approach, and no further details whether the surrounding tissue of the lesion was included or not are reported. Although it might raise ethical concerns, the only way to assess the boundaries of the pathological tissue is by observing its adjacent relationship with a healthy area, thus conserving the surgical intervention along with the sampling collection might not always offer a holistic perspective. Serial sections of the specimens were collected and analysed and the World Health Organisation standardised protocol of histological diagnosis was used. The results after all samples were analysed revealed a higher incidence of granulomas (72%) and a lower incidence of radicular cysts (21.5%), with abscesses and scar tissue being separately considered. Thirteen lesions presented a radiopaque lamina on the preoperative X-ray, but only five of them were histologically diagnosed as cysts, while the rest were epithelialized granulomas. Even though it has been noted that the dimension of the lesion can be related to some extent to the histological diagnosis, the limited sample included in the research does not allow the author to draw an evidence-based conclusion.

The case study published by Ricucci et al. was designed to establish whether the presence of the radiopaque lamina detected on preoperative periapical X-ray was linked to the histopathological diagnosis [15]. Even though the sample size was not substantial, the design of the study was coherently conducted. The methodology was thoroughly described and comprehensive imaging, surgical and histopathological protocols were presented. Fifty-seven teeth with periapical lesions were assessed using paralleling radiographs and were subjected to extraction. Conventional manoeuvring was used for the histopathological samples from the moment of extraction to the time of microscopic assessment. Serial sections were analysed for a more accurate diagnosis. Although not a large sample, the results reinforce other reports published in textbooks [86,87]; hence, the authors appraise the representative sample size, considering the statements that were well founded. Ten out of 57 (18%) of the lesions were histologically diagnosed as cysts, 35 cases were diagnosed as granulomas and the rest being abscesses [15]. Comparing the two diagnostic tools, the results are intuitive. Only 3 out of 10 radiological imaging presenting radiopaque lamina were cysts, while 7 out of 47 lesions not presenting a cyst-like imaging characteristic were in fact histopathologically confirmed as being cysts. The discussion and conclusion were related with the results derived from this study and other relevant articles published, and all stating that radiological appearance cannot be predictable in association with the histological findings [15,77]. Moreover, the authors advise the clinicians and researchers to avoid imaging interpretation using “cyst” or “granuloma” terminology, nonetheless “periapical radiolucent lesion” is less-specific and pertinent with regard to the true unknown histological diagnosis.

Samples were directly immersed for ≥48 h in 10% buffered formalin. For the next 3–4 weeks, samples were demineralised in a solution of formic acid (22.5% vol/vol) and sodium citrate (10% wt/vol) that was constantly agitated. Samples were then rinsed with water for 24 to 48 h and dehydrated. In toto embedment procedure was next performed, oriented parallel to the long axis of the root, followed by serial sectioning and slides staining with hematoxylin and eosin.

A study by Ricucci et al. concluded that the presence of bacterial biofilms is subject to persistent chronic pathology that includes large periapical lesions and cysts [79]. The study contained detailed information about the materials and methods used and standardised tissue processing, including periapical imaging investigation for clinical assessment. The aim of the study was to analyse the presence of intra- and extracanal biofilm on treated and untreated teeth, but also to examine possible connections between the bacterial biofilm and radiographic size of the lesion as well as histopathological diagnosis. For the second purpose, statistical analysis was carried out using both Fisher and chi-square tests to determine any associations between the above described parameters. The periapical lesions were split into two categories, one smaller and equal to 5 mm and the other one larger than 5 mm, while histological diagnosis was described as granulomas, cysts and abscesses. The results were obtained for 71 samples with available radiographic investigation, concluding that 62% of lesions ≤5 mm contained bacterial biofilms, whereas 82% of the lesions >5 mm were identified with the same condition. Untreated canals were separately analysed, and the outcome revealed the presence of bacterial biofilm in 59% of the cases with small lesions (≤5 mm) and 87.5% of cases with lesions larger than 5 mm. Unfortunately, there is no other conclusion or result in this study that links the clinical diagnosis based upon imaging findings and the histopathological assessment, the authors focusing on the biofilm’s presence in different clinical and histopathological situations.

The correlation between the histopathological diagnosis and the viewpoint derived from the interpretation of periapical imaging has been questioned and studied since early research [80]; however, the absence of a systematic analysis allowed ambiguous interpretations and divided opinions. This article presents a series of 101 cases for which the correct correlation between the biological diagnosis and the X-ray assessment was successful in 12.7% of cysts and 58.7% of granulomas. The results lead to the conclusion that periapical imaging should be used only for locating the pathological process and clinicians should not guide their clinic approach considering an image feature; moreover, textbooks describing roentgenographic characteristics differentiating cyst from granuloma should review their statements.

A comparative study published in 2006 developed at King’s College London Dental Institute assessed the diagnostic capacity of three imaging tools (ultrasound, conventional and digital periapical radiography) on a group of 15 patients presenting apical pathology on upper and lower anterior teeth requiring apicoectomy [81]. The results of the study strengthened previous publications [46,88] regarding the accuracy of ultrasonography investigation in differentiating apical cyst from granuloma, reporting a 100% agreement to the histopathological diagnosis [81].The measurements obtained from the conventional X-rays disagreed with the results acquired from the digital imaging technique; however, both tools were unable to differentiate the nature of the endodontic lesion.

A previous research evaluating 396 endodontic lesions reported a correct correlation between the preliminary radiographic diagnosis and the histopathological confirmation in 81% of the cases with granuloma while for the cyst cases the consensus was 48% [82]. Despite the fact that for this publication the number of correctly interpreted periapical X-rays is higher than in other studies, the authors conclude that cysts cannot be differentiated from granulomas only by using periapical radiographic investigations.

A retrospective study calculates the prevalence of cysts in connection to the dimension of the pathological process by measuring the area of 69 lesions present on periapical X-rays, confronting the results with the histological diagnosis [83]. The results of this research present a higher incidence (92%) of periapical cysts in lesions measuring >200 mm^2^ comparing to lesions <50 mm^2^ where the incidence drops to 20% for cysts while chances of detecting a granuloma are 80% [83]. The authors recommend retrograde surgical approach in all cases where the area of the pathological process measures more than 200 mm^2^ for the high prevalence of cysts in these cases.

A study published in 1968 studied 41 periapical lesions by injecting a water-soluble contrast medium through the prepared root canal to observe if any relationship can be noticed between the periapical image acquired after the contrast agent was injected and the histological diagnosis [84]. There were no specific dissimilarities found produced by the contrast medium on the periapical X-ray that could allow an imaging distinction between granuloma, cyst or chronic abscess; hence, no relationship between the histologic and the roentgenologic diagnosis was reported.

## 4. Discussion

This review is based on strict inclusion and exclusion criteria that were designed to deliver an overview of published materials on the subject. The constraint is represented by the limited number of included articles and samples investigated by each paper.

Reviewing the literature and evaluating whether a clinical diagnosis can be related to the histological diagnosis using periapical X-ray requires agreed terminology, which has been used in this review. It is disorienting even for skilled researchers to find divergent classifications of the same pathology, some published in articles and books. As a result in many cases, academic staff, researchers and books teach undergraduate students histological diagnoses and the tools to ascertain them clinically. For the moment, clinical tools available do not translate perfectly into the true histological status of the tissue; thus, various clinical diagnoses have been published for the same clinical presentation in the literature over time. The clinical approach available for the treatment of periapical pathology is limited whatever the histological assessment; thus, it is important for clinicians to understand and clearly make the distinction between the two assessments in order to properly guide their clinical approach.

In an attempt to address this, the American Association of Endodontists (AAE) held a conference in 2008 that aimed to generate a consensus in clinical endodontic diagnosis [89]. Foreseeing the need for guidelines and checklists in Endodontology, the AAE and the American Board of Endodontics proposed the acceptance of terminology aiming not only to standardise definitions for all people working in or related to this field (students, dentists, researchers, professors, experts, endodontists, third parties, etc.) but also to translate other difficulties generated by the absence of a universal language (test interpretations, clinical and paraclinical assessments, research criteria, etc.). This review has used the terminology published by the AAE for the appraisal of clinical diagnosis and Nair’s [90] classification for the histological evaluation. Table 4 shows the terminology used for clinical diagnosis of the periapical tissue published after the 2008 AAE conference.

Many publications have correlated the true histological status of the periapical tissue with clinical signs and symptoms to translate and link the cellular world to daily practice [26,51,52,73,78]. Unfortunately, the available clinical tools that allow the clinician to diagnose do not truly assess histopathology; therefore, the only use of this hybrid classification is to induce a more subjective approach.

Nair [90] published a clinical report on 256 lesions from human extracted teeth and developed a classification system based only upon histopathological findings. Table 5 describes Nair’s histopathological classification.

There may exist an argument for the irrelevance of determining the exact histological status of periapical tissue since the advocated treatment in each case is the same. Assuming one would know whether the lesion is a true or pocket cyst, or could differentiate between epithelialized or non-epithelialized granuloma, it would not change the clinical approach since either surgical or nonsurgical endodontic retreatment would be opted for. Researchers and clinicians try to translate and link the microbiology to the patient’s clinical status, still the results are two distinct classifications hard to be pragmatically connected. The correct assessment should guide to a more or a less invasive approach: orthograde or retrograde endodontic retreatment.

**Figure 2:**
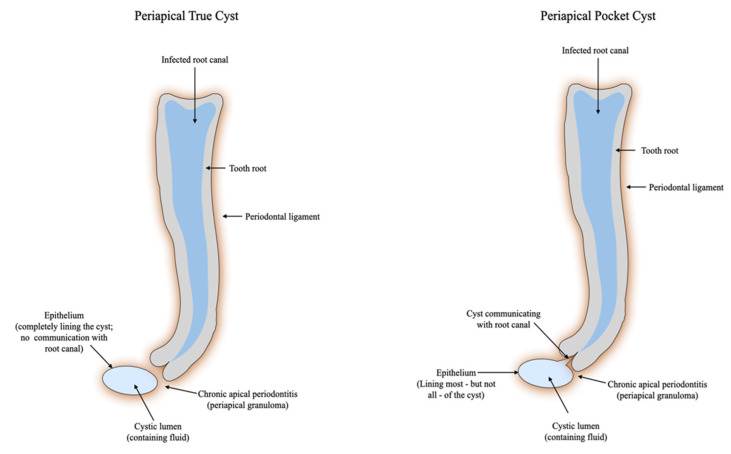
Schematic of histological features of a periapical pocket and true cyst [92]. Lin et al. deduced that the reason for cysts causing failure in endodontics is not yet established. In their study, 9 out of 29 teeth with endodontic treatment and histological diagnosis of periapical cyst did not show periapical imaging changes at the time the conventional RCT was performed [42]. The study does not mention whether there was a coherent protocol carried out by trained specialists, and since the absence of periapical radiolucencies does not certify the absence of a periapical pathology [93,94], their conclusions may be spurious. On the other hand, the presence of periapical radiolucencies neither certifies an existing disease, but according to other studies, these imaging findings can also mark the presence of a scar tissue that does not require further treatment [95,96].

In contrast to the previous statement, the next study [28] refers to the necessity of a more detailed differential diagnosis between true and pocket cyst, considering, together with other publications [43,56,57,58,66,91], that there is a chance for a pocket cyst to heal after orthograde treatment. The same author suggests that true cyst could heal as well if the apoptosis mechanism is elicited by the endodontic re/treatment [28]. Schematics of the histological aspects of epithelialized and non-epithelialized granuloma, a periapical pocket cyst and a periapical true cyst are shown in Figure 2.

A recent study concluded that the most frequent cause of endodontic failure followed by extraction is inappropriate restorative treatment [97]. The purpose of this study, however, was not to examine the healing of cysts but to highlight the available evidence that periapical imaging does not possess predictable features for granulomas and/or for cysts and one cannot ascertain by only X-ray the need of surgical intervention.

Persistent apical pathology is a controversial subject and it is influenced by the following factors: refractory intracanal infection, hosts immune response to direct or non-specific injury, foreign body reaction, extraradicular infection or presence of a cystic lesion [55,98]. The aetiology described is of endodontic origin and clinicians must be aware of all the variables that influence the long-term outcome of an endodontic treatment, and comprehensive assessment and approach should be opted for. The incidence of cyst among periapical lesions ranges from 6% to 55%. However, bearing in mind that serial section histology is required, the actual numbers drop to 15% incidence, thereby limiting its relevance [15].

A published study shows that after X-ray assessment General Dental Practitioners have misdiagnosed 45.9% of the cases, close to endodontists with 42.2% [99]. Hence, the “daughter test” approach should be carefully applied when pondering apicoectomy or extraction as preferred treatment plan. There is no evidence-based publication showing that pocket or true cysts do not heal after orthograde endodontic treatment; moreover, it is impossible to prove otherwise before a sensitive tool for non-invasive periapical assessment is available.

Ultrasound real-time imaging investigation is a promising tool for periapical differential diagnosis, yet limited research is available. Some studies show a remarkable accuracy (86.7–100%) when comparing it to the gold standard. However, the sample size analysed cannot be considered scientifically conclusive (*n* = 11, *n* = 15, *n* = 20) [46,100,101]. The power of a conclusive research is governed by the *n* value that directly influences the Confidence Level and the Margin of Error (the two define the level of precision), thus empowering the results and conclusions [102]. However, no single index should replace the logic behind scientific research [103]. The external validity of ultrasound studies should be considered, due to limits in General Practitioner knowledge and availability to the required machinery.

Considering that all the studies assessed were conducted in strict conditions, it is idealistic to believe a better performance could be achieved in private practice, where clinicians face the situation of reading X-rays from different sources with variable quality. There are so many variables in dental X-ray interpretation that it is impossible even for the most trained specialists to rely completely on the information assessed; hence, CBCT has been introduced, bearing in mind that even 3D imaging can present with significant discrepancies in terms of quality and diagnostic value. However, for the differential diagnosis between cyst and granuloma, no current imaging tool, available for clinical practice use, is as accurate as histopathology.

A study assessing the diagnostic accuracy of periapical X-ray and CBCT on the diagnosis of apical periodontitis concludes that both imaging methods have comparable sensitivity and positive predictive value [34]. Histopathology was used to confirm the periapical diagnosis for the 86 roots examined in the article cited above.

An earlier research explains that periapical imaging does not disclose periapical pathology unless the cortical bone is involved in the lesion, undervaluing the real histopathological diagnosis [104]. Many clinicians assign the presence of a radiopaque lamina or of sclerotic border as a pathognomonic imaging sign in the diagnosis of apical cyst. The evidence cited in this review does not only prove that the specific feature mentioned is not sufficient to reach diagnosis of a cyst [26], but it also reinforces the idea that caution should be taken by clinicians when assessing not only periapical X-rays but also CBCT investigation [9].

A previous study that performed a review of 1108 cases searching for the histopathological nature of the periapical pathology found only 16.8% lesions as being cystic [91]. It is crucial to be recalled that the study’s histological protocol in diagnosing a lesion as a cyst was including any sample that contained epithelial lining, even if it was fragmentary. Lesions larger than 6 mm in diameter containing a fibrous capsule with inflammatory infiltration resembling to an infected cyst with a destroyed epithelial lining were also classified as cysts; however, the paper’s resulting incidence of this pathology is compelling.

On the basis of another study evaluating the correlation of internal apical resorption and the type of the apical lesion on 102 specimens, only 20% was found to be the incidence of cystic lesions [105].

## 5. Conclusions

Although the aim of this review was not to establish guidelines for clinical approaches, it does help to provide a more comprehensive assessment of all endodontic clinical situations to avoid overtreatment and suggesting conservative and minimal invasive approaches. In support to the previous statement, the 2006 study conducted at King’s College London indicates that the radiological diagnosis is not accurate; hence, the process should be improved [15].

Overdiagnosis gives rise to overtreatment; therefore, periapical diagnosis is crucial for the decision making of the appropriate treatment [106]. It is a limitation of any diagnostic tool. Imaging investigation is the most commonly used in periapical pathology despite not being the gold standard for differential diagnosis. Nonsurgical approach is successful in 84.4% of the cases [107] and thus can be opted for as first line of treatment. Moreover, the same prospective study determined that only 35.7% of the unhealed periapical lesions were cysts [107].

There is no doubt that periapical X-ray is not a precise diagnostic tool and subjective imaging features cannot differentiate between the type and nature of the periapical lesion (i.e., cyst or granuloma or a diffuse apical periodontitis).

This review clearly presents all the aspects needed for a Clinician to understand that, unless retrograde approach represents the least invasive option, endodontic re/treatment and follow-up is the preferrable approach to treat periapical lesions.

Persistent apical pathology, symptomatology and clinical findings are fundamental aspects to be followed-up. Persistence of pathology could call for endodontic surgery, but this should happen after the orthograde re/treatment has been performed [108].

In contemporary practice, many treatment options are available for failed primary root canal therapy including apicoectomy, re/transplantation and extraction followed by implant/fixed prosthesis or extraction without further restoration [109]. Although the patient’s decision and patient-related outcomes influence the chosen approach, all possibilities should be comprehensively explained by the GDP, including the situations where limited training/skills, lack of experience or self-confidence warrant specialist referral.

Further well-designed research is needed to complement the available published materials. Considering the reviewed subject, awareness campaigns would be appreciated and should help the Clinicians understand the limitations of periapical X-ray in endodontic diagnosis and develop coherent treatment plans without misconceptions. Furthermore, the diffusion of CBCT as a diagnostic tool will cascade also on the treatment planning in endodontics, leading to a more conservative approach.

## Figures and Tables

**Table 4:** Diagnostic terminology used for clinical assessment of the periapical status [89].

Normal Apical Tissues	Teeth with Normal Periradicular Tissues That Are Not Sensitive to Percussion or Palpation Testing. The Lamina Dura Surrounding the Root Is Intact, and the Periodontal Ligament Space Is Uniform.
Symptomatic apical periodontitis	Inflammation, usually of the apical periodontium, producing clinical symptoms including a painful response to biting and/or percussion or palpation. It might or might not be associated with an apical radiolucent area.
Asymptomatic apical periodontitis	Inflammation and destruction of apical periodontium that is of pulpal origin, appears as an apical radiolucent area, and does not produce clinical symptoms.
Acute apical abscess	An inflammatory reaction to pulpal infection and necrosis characterized by rapid onset, spontaneous pain, tenderness of the tooth to pressure, pus formation and swelling of associated tissues.
Chronic apical abscess	An inflammatory reaction to pulpal infection and necrosis characterized by gradual onset, little or no discomfort, and the intermittent discharge of pus through an associated sinus tract.
Condensing osteitis	Diffuse radiopaque lesion representing a localized bony reaction to a low-grade inflammatory stimulus, usually seen at apex of tooth.

**Table 5:** Histopathological classification of the periapical pathology.

Periapical Abscess	Periapical Granuloma	Periapical Cyst
Epithelialized abscess	Epithelialized granuloma	Apical true cyst
Non-epithelialized abscess	Non-epithelialized granuloma	Apical pocket cyst

## Data Availability

The data that support the findings of this study are available from the corresponding author, (F.F.), upon reasonable request.
